# HSP110 as a Diagnostic but Not a Prognostic Biomarker in Colorectal Cancer With Microsatellite Instability

**DOI:** 10.3389/fgene.2021.769281

**Published:** 2022-01-03

**Authors:** Gaelle Tachon, Arnaud Chong-Si-Tsaon, Thierry Lecomte, Audelaure Junca, Éric Frouin, Elodie Miquelestorena-Standley, Julie Godet, Camille Evrard, Violaine Randrian, Romain Chautard, Marie-Luce Auriault, Valérie Moulin, Serge Guyetant, Gaelle Fromont, Lucie Karayan-Tapon, David Tougeron

**Affiliations:** ^1^ Faculté de Médecine, Université de Poitiers, Poitiers, France; ^2^ INSERM U-1084, Laboratoire des Neurosciences Expérimentales et Cliniques Université de Poitiers, Poitiers, France; ^3^ Laboratoire de Cancérologie Biologique, Centre Hospitalo-Universitaire de Poitiers, Poitiers, France; ^4^ Service d’Anatomopathologie, Centre Hospitalo-Universitaire de Poitiers, Poitiers, France; ^5^ Inserm UMR 1069, Nutrition, Croissance et Cancer, Université de Tours, Tours, France; ^6^ Service de Gastroentérologie, Centre Hospitalo-Universitaire de Tours, Tours, France; ^7^ Service d’anatomopathologie, Centre Hospitalo-Universitaire de Tours, Tours, France; ^8^ Service d’oncologie Médicale, Centre Hospitalo-Universitaire de Poitiers, Poitiers, France; ^9^ Service de Gastroentérologie, Centre Hospitalo-Universitaire de Poitiers, Poitiers, France; ^10^ Service de Gastroentérologie, Centre Hospitalier de la Rochelle, La Rochelle, France; ^11^ Service d’Oncologie Médicale, Centre Hospitalier de la Rochelle, La Rochelle, France

**Keywords:** colorectal cancer, microsatellite instability, deficient mismatch repair, HSP110, adjuvant chemotherapy, biomarker, Lynch syndrome

## Abstract

Determination of microsatellite instability (MSI) using molecular test and deficient mismatch repair (dMMR) using immunohistochemistry (IHC) has major implications on colorectal cancer (CRC) management. The *HSP110 T*_*17*_ microsatellite has been reported to be more monomorphic than the common markers used for MSI determination. Large deletion of *HSP110 T*_*17*_ has been associated with efficacy of adjuvant chemotherapy in dMMR/MSI CRCs. The aim of this study was to evaluate the interest of HSP110 deletion/expression as a diagnostic tool of dMMR/MSI CRCs and a predictive tool of adjuvant chemotherapy efficacy. All patients with MSI CRC classified by molecular testing were included in this multicenter prospective cohort (*n* = 381). IHC of the 4 MMR proteins was carried out. HSP110 expression was carried out by IHC (*n* = 343), and the size of *HSP110 T*_*17*_ deletion was determined by PCR (n = 327). In the 293 MSI CRCs with both tests, a strong correlation was found between the expression of HSP110 protein and the size of *HSP110 T*_*17*_ deletion. Only 5.8% of MSI CRCs had no *HSP110 T*_*17*_ deletion (*n* = 19/327). *HSP110 T*_*17*_ deletion helped to re-classify 4 of the 9 pMMR/MSI discordance cases as pMMR/MSS cases. We did not observe any correlation between HSP110 expression or *HSP110 T*_*17*_ deletion size with time to recurrence in patients with stage II and III CRC, treated with or without adjuvant chemotherapy. HSP110 is neither a robust prognosis marker nor a predictor tool of adjuvant chemotherapy efficacy in dMMR/MSI CRC. However, *HSP110 T_17_* is an interesting marker, which may be combined with the other pentaplex markers to identify discordant cases between MMR IHC and MSI.

## Introduction

Approximately 15% of colorectal cancers (CRCs) have microsatellite instability (MSI) due to the deficient mismatch repair (dMMR) system (Umar et al., 2004; Evrard et al., 2019). Determination of MMR/MSI status is a key issue in the management of CRC patients. dMMR/MSI status is associated with Lynch syndrome but is also a prognostic and predictive factor of sensitivity to chemotherapy and immune checkpoint inhibitors. Indeed, dMMR/MSI status is associated with good prognosis in non-metastatic CRC, particularly for patients with stage II CRC, which in most cases does not require adjuvant chemotherapy (Sargent et al., 2010). Moreover, dMMR/MSI status has been associated with chemoresistance to adjuvant 5-fluorouracil (5-FU) in stage II CRC (Tougeron et al., 2016). Finally, the high rate of mutational load in dMMR/MSI tumors leads to multiple tumor-specific neo-antigens recruiting cytotoxic T-cells, which explain both the good prognosis and the high sensitivity to immune checkpoint inhibitors (Tougeron et al., 2009; Le et al., 2015; André et al., 2020).

HSP110 protein belongs to the family of large heat shock proteins (HSPs) (Zuo et al., 2016). In CRC, HSP110 promotes the proliferation of tumor cells by activating signal transducer and activator of transcription 3, STAT3 (Berthenet et al., 2017). In 2011, Dorard et al. identified *HSP110* as a target of microsatellite instability (Dorard et al., 2011). In the intron 8 of *HSP110*, there exists a region of mononucleotide repeats of thymine, which is located upstream of the splice acceptor-site of exon 9. This region is well conserved across species with only two known alleles, 16 repeats (T_16_) and 17 repeats (T_17_), the latter being more frequent. The *HSP110 T*_*17*_ marker has been reported to be more monomorphic than the common mononucleotide markers used for the determination of MSI status (Buhard et al., 2016; Berardinelli et al., 2018; How-Kit et al., 2018). Indeed, about 97% of dMMR/MSI CRCs presented 1 to 7 base pairs (bp) deletion within the T_17_ region. These deletions, when they were bi-allelic and of large size (≥5 bp), led to complete inactivation of the HSP110 protein (Dorard et al., 2011). Large deletion of *HSP110 T*_*17*_ has been associated with efficacy of adjuvant chemotherapy, 5-FU alone or with oxaliplatin, in non-metastatic stage II and III dMMR/MSI CRCs by one team (Dorard et al., 2011; Collura et al., 2014). By contrast, neither significant survival difference nor impact on adjuvant chemotherapy efficacy between large and small *HSP110 T*_*17*_ deletions was observed in another study (Kim et al., 2014).

Kim JH et al. analyzed HSP110 expression by immunohistochemistry (IHC) in dMMR/MSI CRCs, and low expression of HSP110 was significantly associated with deletion of 4 bp or more of *HSP110* thymine repeats (Kim et al., 2014). High expression of HSP110 was observed in normal tissue, pMMR/MSS CRCs and dMMR/MSI CRCs with short *HSP110 T*_*17*_ deletion. Low HSP110 expression was associated with better disease-free survival (DFS) but not the size of *HSP110 T*_*17*_ deletion. These results and other studies suggested discordant results concerning HSP110 expression by IHC and/or size of *HSP110 T*_*17*_ deletion as a prognostic factor and/or predictor of adjuvant chemotherapy efficacy in stage II and III dMMR/MSI CRCs (Dorard et al., 2011; Collura et al., 2014; Kim et al., 2014; Oh et al., 2017). dMMR/MSI CRCs have been associated with resistance to adjuvant 5FU, mostly in stage II CRC, as compared to pMMR/MSS tumors. Nevertheless, these studies have suggested that among dMMR/MSI, *HSP110 T*_*17*_ deletion size could be associated with sensitivity to adjuvant 5FU, a finding suggesting that non-metastatic dMMR/MSI CRC could have different levels of chemoresistance to 5FU due to *HSP110 T*_*17*_ deletion.

The objective of this study was to evaluate the correlation between *HSP110 T*_*17*_ deletion and dMMR/MSI status identified by standard procedures. We also evaluated both *HSP110 T*_*17*_ deletion and HSP110 expression as prognostic factors or predictors of response to adjuvant chemotherapy in dMMR/MSI CRCs.

## Patients and Methods

### Patients

From 2003 to 2015, all patients with a CRC classified as “MSI” by molecular testing (Pentaplex panel) from the cancer biology departments of the Poitiers and Tours University Hospitals were included in this multicenter prospective cohort (n = 381). Proficient MMR (pMMR) or dMMR statuses were determined secondarily (MLH1, PMS2, MSH2, and MSH6 protein expression). Most MMR IHC and MSI molecular tests were carried out in routine clinical practice. All cases, including discordant cases (pMMR/MSI), were evaluated in light of *HSP110 T*_*17*_ deletion results.

The study has been approved by the ethics committees of the Poitiers and Tours University Hospitals (Comité de protection des personnes Ouest III, n DC-2008-565 and n 2018-039). The study was performed according to the principles of the Declaration of Helsinki.

Main patients (gender and age) and tumor (tumor site, TNM stage, VELIPI criteria (vascular emboli, lymphatic invasion, or perineural invasion), tumor grade, tumor perforation, and initial bowel obstruction) characteristics were collected. Germline Lynch syndrome versus sporadic dMMR/MSI cases was determined, as previously described (Tougeron et al., 2020). Briefly, the molecular mechanism underlying the MMR deficiency was based on the detection of MMR gene germline mutation, Amsterdam II criteria, MMR protein expression, *BRAF* status, and *MLH1* promoter methylation.

### Determination of MSI and MMR Status

Tumor DNA was extracted in routine practice from formalin-fixed paraffin-embedded tumor tissue using a KAPA Express Extract© kit (ROCHE, Basel, Switzerland). The same tumor DNA was also used to determine *KRAS*, *NRAS*, and *BRAF* mutations using next-generation sequencing.

MSI phenotype was assessed by analyzing microsatellite loci consisting of 5 mononucleotide markers, BAT-25, BAT-26, NR21, NR24, and NR27 (Kit PROMEGA, ref MD1641, Madison, Wisconsin, United States), as previously described (Pentaplex panel) (Buhard et al., 2006; Tachon et al., 2018). Briefly, PCR fragments were separated according to their size by capillary electrophoresis using an ABI 3500Dx Genetic Analyzer© (Applied Biosystems, Foster City, United States). The results were analyzed using GeneMapper v4.1© software (Applied Biosystems) and interpreted by expert molecular biologists. MSI was defined by the presence of instability affecting at least 3 of the 5 markers. In the case of one or two markers with instability, a comparative analysis of normal colon tissue and tumor DNA was performed, as polymorphisms have been reported, especially in African ethnicities (Augustus and Ellis, 2018).

For all cases, both tissue microarray and whole tissue sections were available. For tissue microarray construction, nine biopsy cores of 0.6 mm diameter per patient (3 in tumor center, 3 in invasive front, and 3 in non-tumor tissue) have been performed using Alphelys^©^ plateform (MTA Booster^©^ version 1.01, Plaisir, France).

For determination of MMR status, most IHCs were carried out in routine clinical practice from formalin-fixed paraffin-embedded tumor tissue using antibodies directed against MLH1 (clone M1), MSH2 (clone G219-1129), MSH6 (clone 44), and PMS2 (clone EPR3947) proteins (Ventana Medical Systems, Tucson, AZ, United States), as previously described (Tachon et al., 2018; Singh et al., 2021). If IHC was not performed or was discordant with the MSI test (pMMR/MSI), a new MMR IHC was performed on tissue microarray. A tumor was considered dMMR if it presented loss of nuclear staining of at least one of the four MMR proteins.

### HSP110 Immunohistochemistry

HSP110 IHC was carried out on whole-section tissues with the antibody directed against the C-terminus part of HSP110 protein (Leica Biosystems, NCL-HSP105, RRID:AB_563775, Newcastle upon Tyne, United Kingdom), as described by Kim et al. (2014). This HSP110 antibody is designed to target only wild-type (wt) HSP110 protein and therefore does not recognize the truncated HSP110 protein caused by *HSP110* intronic T_17_ deletions. The staining was mostly cytoplasmic and sometimes associated with nuclear staining, as previously described (Kim et al., 2014). The same intensity grading as that of Oh et al. was used (Oh et al., 2017), with four-tier intensity scores ranging from 0 (no expression) to 3 (stronger nuclear-to-cytoplasmic expression than the internal positive control) (Supplementary Figure S1). Internal non-tumor tissue, precisely normal colonic mucosal epithelial cells and/or lymphocytes, served as the positive control. The intensity score was considered when at least 5% of the tumor surface was stained. For each sample, the most represented score occupying the largest area of the tumor, called the “dominant intensity score” by Oh *et al.*, was determined (Oh et al., 2017) (Supplementary Figure S2). Nuclear positivity was defined as nuclear staining of at least 10% of the tumor cells.

Two independent expert pathologists, unaware of other results and *HSP110 T*_*17*_ deletion, conducted the scoring in the 343 formalin-fixed paraffin-embedded samples available for HSP110 expression IHC. Conflicting results between the two observers were reviewed and discussed, and a consensus was reached. The tumor was classified as HSP110 “high” when the dominant intensity score was 2 + or 3+ and HSP110 “low” when the dominant intensity score was 0 or 1+.

### Molecular Determination of *HSP110 T*_*17*_ Deletion

**FIGURE 1:**
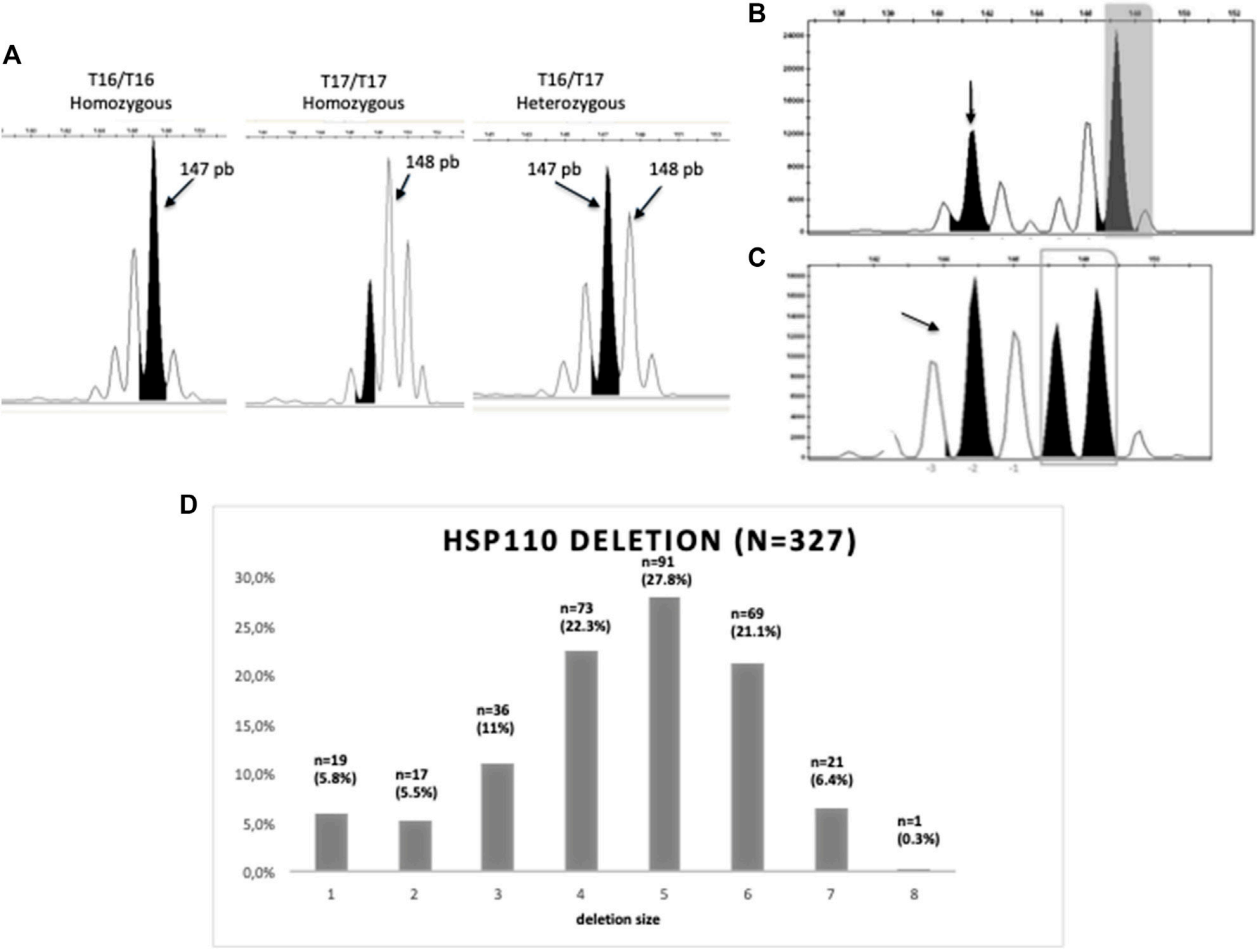
Molecular determination of *HSP110 T*_*17*_ deletion. **(A)** Homozygous *T*_*16*_ */T*_*16*_ and *HSP110 T*_*17*_ */T*_*17*_ and heterozygous *T*_*16*_ */T*_*17*_ HSP110 profiles from MSS CRC tissues. Peak of 147bp is indicated in black. **(B)** MSI tumor with a large deletion (−5bp) of *HSP110 T*_*17*_. **(C)** MSI tumor with a small deletion (−2bp) of *HSP110 T*_*17*_. The gray frame is the polymorphic area. Arrows indicate the size of the deleted transcript. **(D)** Distribution of samples according to the size of *HSP110 T*_*17*_ deletion.

The size of *HSP110 T*_*17*_ microsatellite deletion was determined by PCR followed by capillary electrophoresis on the 327 tumor DNA available. To amplify the region of interest, homemade primers (forward, 5′-TAMRACCCTGTCCATCCATTGGAATTGA-3′; reverse, 5′-GGA​ACT​GCA​TCT​GTG​ACG​GAA-3′) and standard PCR procedure (initial denaturation step at 94°C for 10 min, then 40 cycles at 94°C for 30 s, 57°C for 30 s, and 72°C for 1 min) were used. Results were interpreted by two independent molecular biologists using GeneMapper v4.1^©^ software (Applied Biosystems, Foster City, United States). To determine the polymorphic zone corresponding to the T_16_/T_16_, T_16_/T_17_, and T_17_/T_17_ genotypes (Dorard et al., 2011), 36 MSS CRCs were analyzed. The polymorphic zone corresponded to 147–148 base pairs (bp) fragment sizes, 147 bp for the T_16_ allele and 148 bp for the T_17_ allele (Figure 1A). Information about homozygous T_16_ or T_17_ or heterozygous T_16_/T_17_ was not collected in this work, in which only the polymorphic area was established.

Deletions on the *HSP110 T*_*17*_ microsatellite resulted in peaks smaller than the polymorphic zone of 147 bp (Figures 1A–C). For the majority of the analyzed MSI tumor samples, only one mutated allele type was detected, corresponding to the main clonal population present in the tumors, as described by Collura et al. (2014). For cases that displayed multiallelic profiles, the peak associated with the larger *T*_*17*_ deletion, which did not appear to result from the Taq polymerase stuttering, was considered. Two different thresholds for the scoring of *HSP110 T*_*17*_ deletion were used according to previous publications, 5 bp (Collura et al., 2014) and 4 bp (Kim et al., 2014). For all analyses, 5 bp was used first as the primary endpoint.

Two independent expert molecular biologists, unaware of each other’s results and IHC HSP110 results, conducted the molecular interpretation. Conflicting results between the two observers were reviewed and discussed, and a consensus was reached.

### Statistical Analysis

Follow-up was calculated by reverse Kaplan–Meier estimation. Some patients with sporadic MSI CRC were elderly with comorbidities and could die from causes other than CRC; consequently, time to recurrence (TTR) and disease-free survival (DFS), cancer-specific survival (CSS), and overall survival (OS) were determined. For non-metastatic patients with curative surgery, TTR was defined as the time between surgery and the date of recurrence or death from the same cancer, whichever occurred first (Birgisson et al., 2011). DFS was defined as the time between curative surgery and the date of recurrence or death, whatever the cause and whichever occurred first. OS was defined as the time between CRC diagnosis and the date of death, whatever the cause. Alive patients were censored at the date of last assessment. CSS was defined as the time between CRC diagnosis and the date of death from the same cancer. Alive patients without recurrence were censored at the date of last assessment. Survival curves were estimated using the Kaplan–Meier method and compared using the log-rank test.

Variables with *p* values of 0.10 or less in univariate analysis were eligible for the Cox multivariable regression model. A *p* value of less than 0.05 was considered statistically significant. All analyses were performed using Statview^©^ software (SAS Institute, Cary, NC).

## Results

### Patient and Tumor Characteristics

A total of 381 patients with MSI CRC were included in this study. The mean age was 69.9 years with 60.1% being women (Table 1). The tumor was localized in the ascending colon in 80.6% of cases and the majority of tumors were stage II or stage III (43.9 and 32.2%, respectively). Tumors were mainly pT3 (60.5%) and moderately or poorly differentiated (43.9 and 44.2%, respectively).

**TABLE 1:** Patient and tumor characteristics.

Characteristics	*n* = 381
Age (mean)	69.9 ± 15.5 years
Gender	
Male	152 (39.9%)
Female	229 (60.1%)
Stage	
0	2 (0.5%)
I	29 (7.7%)
II	165 (43.9%)
III	121 (32.2%)
IV	59 (15.7%)
Missing values	5
Tumor site	
Ascending:	304 (80.6%)
Descending:	58 (15.4%)
Rectum	15 (4.0%)
Missing values	4
Tumor perforation	
Yes	27 (7.5%)
No	333 (92.5%)
Missing values	21
Initial tumor obstruction	
Yes	47 (13.0%)
No	313 (87.0%)
Missing values	21
pT stage	
pTis	2 (0.5%)
pT1	8 (2.2%)
pT2	30 (8.1%)
pT3	225 (60.5%)
pT4	107 (28.7%)
Missing values	9
Tumor grade	
Well differentiated	43 (11.9%)
Moderately differentiated	158 (43.9%)
Poorly differentiated	159 (44.2%)
Missing values	21
Lymph node invasion	
N0	213 (57.1%)
N1	102 (27.4%)
N2	58 (15.5%)
Missing values	8
Mucinous component	
Yes	112 (33.8%)
No	219 (66.2%)
Missing values	50
VELIPI	
Yes	166 (49.8%)
No	167 (50.2%)
Missing values	48
MMR IHC	
Loss of MLH1 and PMS2	299 (79.9%)
Loss of MSH2 and MSH6	45 (12.0%)
Isolated loss of MSH6	10 (2.7%)
Isolated loss of PMS2	7 (1.9%)
Other combinations of MMR protein loss	4 (1.1%)
No loss of MMR proteins	9 (2.4%)
Missing values	7
*MLH1* promoter hypermethylation test (*n* = 112)^a^	
*MLH1* promoter hypermethylation	68 (60.7%)
No *MLH1* promoter hypermethylation	44 (39.3%)
Lynch syndrome	
MMR mutation	29 (8.9%)
Suspected	54 (16.6%)
No	243 (74.5%)
Missing values	55
*RAS* mutation	
Yes	94 (25.0%)
No	282 (75.0%)
Missing values	5
*BRAF* mutation	
Yes	203 (54.0%)
No	173 (46.0%)
Missing values	5

IHC, immunohistochemistry; MMR, mismatch repair; VELIPI, vascular emboli or lymphatic invasion or perinervous invasion.

a *MLH1* promoter hypermethylation was determined in 112 tumors in order to identify sporadic MSI CRCs (tumors with MLH1 loss and no *BRAF* mutation).

*RAS* and *BRAF* mutations were observed in 25.0 and 54.0% of tumors, respectively. Most CRCs had MLH1/PMS2 loss (79.9%) and only 9 (2.9%) were pMMR. *MLH1* promoter hypermethylation was determined in 112 tumors in order to identify sporadic MSI CRCs and was detected in 60.7% (*n* = 68/112). Only 8.9% of patients had a proven germline MMR mutation, and 16.6% had suspected Lynch syndrome.

### Treatments and Outcome

Most patients had a resection of their primary tumors (99.0%). Among patients with non-metastatic tumors (n = 317), six patients had non-curative resection of their tumor and were excluded for survival analyses. Among the stage II and III patients with curative strategy (*n* = 280), 37.0% had received adjuvant chemotherapy, 16.0 and 67.0% in stage II and III, respectively (Table 2). Most adjuvant treatment was 5-FU associated with oxaliplatin (80.6%, *n* = 83/103) and others, fluoropyrimidine alone.

**TABLE 2:** Treatments and outcome in stages II and III with curative surgery.

Stages II (*n* = 165) and III (*n* = 115)	*n* = 280
Adjuvant chemotherapy (n, %)	
Stage II (n = 163)	26 (16.0%)
Stage III (n = 115)	77 (67.0%)
Missing values	2
Recurrence (n, %)	
Stage II (n = 159)	21 (13.2%)
Stage III (n = 114)	27 (23.7%)
Missing values	7
3-year time to recurrence (%)	
Stage II	88.8%
Stage III	76.2%
Median disease-free survival (months)	
Stage II	87.1 ± 10.5
Stage III	76.7 ± 26.4
Median overall survival (months)	
Stage II	91.9 ± 8.2
Stage III	91.5 ± 8.6
5-year cancer-specific survival (%)	
Stage II	88.8%
Stage III	75.0%

Median follow-up of the overall population was 79.2 ± 3.5 months. Overall, 48.3% of patients died, of which 59.2% were cancer-related. Among stage II and III CRC patients with curative surgery, recurrence rates were 13.2 and 23.7%, respectively. Median TTR was not reached. The 3-year TTR rates of stage II and III CRC patients were 88.8 and 76.2%, respectively. Median CSS was not reached. The 5-year CSS rates of stage II and III were 88.8 and 75.0%, respectively.

Concerning patients with metastatic tumors (*n* = 59), median OS and CSS were 12.5 ± 3.8 months and 14.3 ± 3.7 months, respectively.

### HSP110 Expression

Among the 381 patients, 343 formalin-fixed paraffin-embedded samples were available for IHC testing. The most represented intensity score in each tumor, called the “dominant intensity score,” was 3+ (54.2%), followed by 2+ (24.5%) (Supplementary Table S1). A positive HSP110 nuclear staining was found in 36.4%, mainly in tumors with a 3 + dominant intensity score (*n* = 95) (*p* < 0.001). In addition, a majority of tumors (74.0%) had intratumoral heterogeneity of HSP110 staining, defined by at least two different identified intensity scores. The majority of tumors presented high HSP110 expression (78.7%) defined by a “dominant intensity score” of 2 + or 3+.

### Molecular *HSP110 T*_*17*_ Deletion and MSI Diagnosis

Deletion of *HSP110 T*_*17*_ was determined for 327 MSI CRC. Most samples (*n* = 308, 94.2%) showed deletion of 1–7 bp of the *HSP110 T*_*17*_ microsatellite (Figure 1D). Few tumors had a large *HSP110 T*_*17*_ deletion, 27.8% using a cut-off at 5 bp, 55.6% using a cut-off at 4 bp.

Nineteen cases had no *HSP110 T*_*17*_ deletion (5.8%). When looking at these tumors, they were more frequently located in the descending colon (44.4 versus 13.8%, *p* = 0.002), *BRAF* wt (84.2 versus 43.8%, *p* < 0.001), with no *MLH1* promoter hypermethylation (78.6 versus 32.9%, *p* < 0.001), and all but one had high HSP110 expression. Most of these tumors were MSI/dMMR/no *HSP110 T*_*17*_ deletion (*n* = 15), 14 with MLH1/PMS2 loss and 1 with MSH6 loss, and 4 were MSI/pMMR/no *HSP110 T*_*17*_ deletion. After re-examination and new MSI/MMR IHC testing on new tumor material with special care given to tissue quality and percentage of tumor cells in these 19 cases, the 4 MSI/pMMR/no *HSP110 T*_*17*_ deletion cases were finally considered as MSS with no unstable markers (*n* = 0/5) (Table 3). Regarding the remaining 5 MSI/pMMR/no *HSP110 T*_*17*_ deletion cases, discordance remained despite re-examination and new MSI/MMR IHC tests. None of these patients had MMR germline testing.

**TABLE 3:** Distribution of MMR status according to *HSP110 T*_*17*_ molecular status.

MSI CRC with *HSP110 T*_*17*_ molecular test (n = 327)	*HSP110 T*_*17*_ deletion	*HSP110 T*_*17*_ wt
Before re-examination		
dMMR	303	15
pMMR	5	4
After re-examination		
dMMR	303	15 (MSI/dMMR)
pMMR	5 (MSI/pMMR)	0

MSI, microsatellite instability; CRC, colorectal cancer; MMR, mismatch repair; wt, Wild-type.

Finally, out of 327 cases and after re-examination, 20 were discordant with either MSI/pMMR/*HSP110 T*_*17*_ deletion (n = 5) or MSI/dMMR/no *HSP110 T*_*17*_ wt (n = 15) (Table 3). All in all, *HSP110 T*_*17*_ deletion has a sensitivity of 95.5% to detect dMMR/MSI cases and proves useful in detecting false MSI results.

### Correlation Between HSP110 Expression and *HSP110 T*_*17*_ Deletion

**FIGURE 2:**
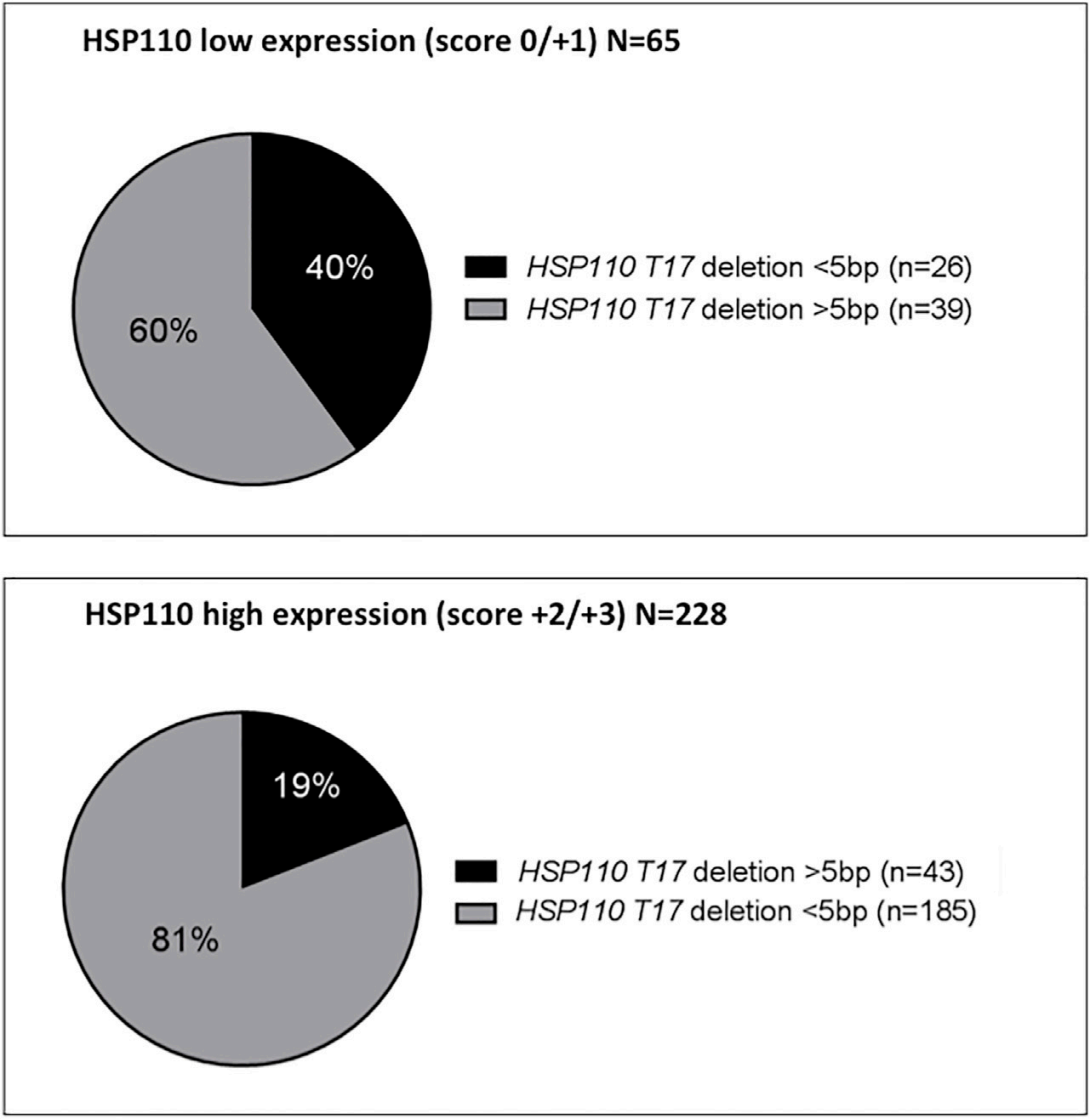
Distribution of the size of *HSP110 T_17_* deletion according to HSP110 immunohistochemistry.

Among the 381 MSI CRCs, 293 were analyzed for HSP110 both by IHC and molecular tests. When comparing HSP110 expression (low versus high) and *HSP110 T*_*17*_ deletion (large versus small) using 5 bp cut-off, a strong correlation was identified (*p* < 0.001), with 76.4% concordance (n = 224/293) **(** Figure 2 **)**. A close result with 59.7% concordance (n = 175/293) was observed using a cut-off of 4 bp (*p* < 0.001). There was no correlation between the presence of nuclear staining and the deletion size of *HSP110 T*_*17*_ using a cut-off at 5 bp (*p* = 0.18) contrary to 4 bp (*p* = 0.01).

### Correlation Between HSP110 Expression/Deletion and Patient/Tumor Characteristics

No correlation was found between HSP110 expression (high versus low) and *HSP110 T*_*17*_ deletion (cut-off at 5 or 4 bp) with main patient characteristics (age, sex, and LS), histopathological features (pTNM stage, tumor site, perforation, initial tumor obstruction, T stage, grade, N stage, mucinous component, and VELIPI criteria), MMR immunohistochemistry, and *BRAF* mutation. By contrast, *RAS* mutations were associated with high HSP110 expression (28.2 versus 12.5%, *p* = 0.006) and small *HSP110 T*_*17*_ deletion using a cut-off at 5 bp (28.0 versus 16.7%, *p* = 0.044). When using a cut-off at 4 bp for *HSP110 T*_*17*_ deletion, a strong correlation with *RAS* mutations was also identified (*p* = 0.003).

### Prognostic and Predictive Values of HSP110 Expression/Deletion

**FIGURE 3:**
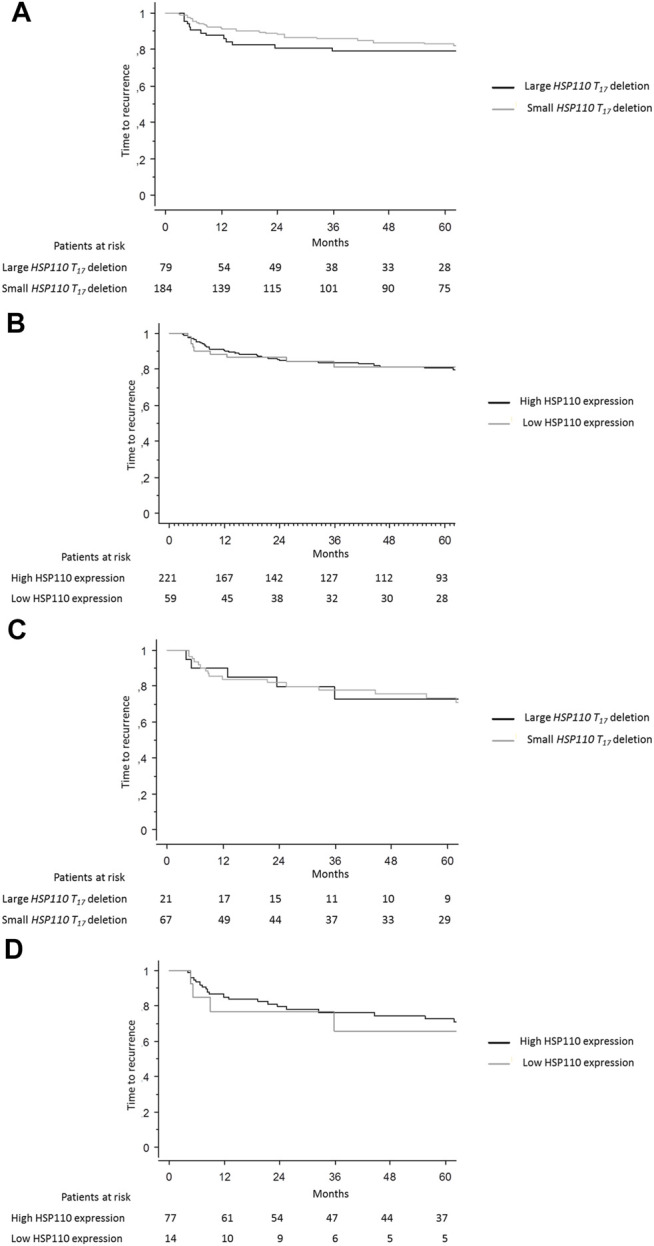
Time to recurrence. Kaplan–Meier curves showing the time to recurrence in patients with stage II or III MSI CRC according to **(A)** size of *HSP110 T*_*17*_ deletion and **(B)** HSP110 expression and time to recurrence in patients with stage II or III MSI CRC who received adjuvant chemotherapy according to **(C)** size of *HSP110 T*_*17*_ deletion and **(D)** HSP110 expression.

In patients with stage II and III MSI CRC, recurrence rates did not differ according to *HSP110 T*_*17*_ deletion size, 19.7% in the large deletion group versus 17.7% in the small deletion group using 5 bp cut-off (*p* = 0.72), or HSP110 expression (*p* = 0.79). TTR and DFS did not differ according to *HSP110 T*_*17*_ deletion size (*p* = 0.45 and *p* = 0.91, respectively) or HSP110 expression (*p* = 0.78 and *p* = 0.64, respectively) (Figures 3A,B). The 3-year TTR rates in the large deletion group versus the small deletion group were 76.7 and 84.1%, respectively. Results were similar when using a cut-off at 4 bp for *HSP110 T*_*17*_ deletion or according to HSP110 nuclear staining.

OS and CSS did not differ according to size of *HSP110 T*_*17*_ deletion using a cut-off at 5 bp (*p* = 0.83 and *p* = 0.29, respectively) or HSP110 expression (*p* = 0.84 and *p* = 0.82, respectively). The 5-year CSS rates in case of large deletion versus small deletion were 79.3 and 83.4%, respectively.

In patients with stage II or III CRC who received adjuvant chemotherapy (*n* = 103), recurrence rates did not differ according to *HSP110 T*_*17*_ deletion size, 23.8% in the large deletion group versus 25.0% in the small deletion group (*p* = 0.91) or HSP110 expression, 28.0% in the high expression group versus 30.8% in the low expression group (*p* = 0.50). The TTR and DFS did not differ according to *HSP110 T*_*17*_ deletion size (*p* = 0.93 and *p* = 0.78, respectively) or HSP110 expression (*p* = 0.78 and *p* = 0.45, respectively) (Figures 3C,D). OS and CSS did not differ according to size of *HSP110 T*_*17*_ deletion (*p* = 0.75 and *p* = 0.49, respectively) or HSP110 expression (*p* = 0.44 and *p* = 0.83, respectively).

Concerning metastatic tumors (n = 59), rates of death did not differ according to *HSP110 T*_*17*_ deletion size (*p* = 0.21) or HSP110 expression (*p* = 0.23), and OS did not differ according to the size of *HSP110 T*_*17*_ deletion (*p* = 0.91) or HSP110 expression (*p* = 0.15).

## Discussion

There were conflicting results concerning the *HSP110 T*_*17*_ microsatellite for MSI determination as compared to the standard method (Pentaplex) but also concerning its prognostic value and its predictive value for adjuvant chemotherapy efficacy in MSI CRCs. Our study is the first to analyze both the expression of HSP110 and the size of the deletion of the *HSP110 T17* microsatellite in a large multicenter study of 381 MSI CRCs. We identified a strong correlation between expression of HSP110 protein and size of deletion of *HSP110 T*_*17*_. With 95.5% sensitivity to identify dMMR/MSI CRCs**,** *HSP110 T*_*17*_ performance seems similar to actual standard tests, namely, MMR IHC and MSI molecular testing. That said, we did not observe any prognostic value or predictive value of adjuvant chemotherapy efficacy of HSP110 expression or deletion size of the *HSP110 T*_*17*_ microsatellite.

In our study, we performed both molecular testing and MMR IHC for MSI determination. In most studies on HSP110, dMMR/MSI CRCs were defined according to either MMR IHC or molecular testing but not both (Dorard et al., 2011; Collura et al., 2014; Buhard et al., 2016; Oh et al., 2017; Berardinelli et al., 2018; How-Kit et al., 2018). This important point is a major limitation to the evaluation of *HSP110 T17* deletion as a diagnostic tool for MSI determination. MMR IHC presents sensitivity between 85 and 100% and specificity between 85 and 92% (Zhang and Li, 2013; Snowsill et al., 2017). Sensitivity and specificity of the Pentaplex panel range between 90 and 100% (Xicola et al., 2007; Goel et al., 2010). Moreover, some studies have shown discordance between MMR IHC and MSI molecular testing, ranging from 1 to 10% (Lindor et al., 2002; Watson et al., 2007; Jaffrelot et al., 2019; Guyot; De Salins et al., 2021). In our cohort, all tumors were initially determined as MSI using a robust test (Pentaplex panel) (Suraweera et al., 2002; Wong et al., 2006). Most tumors had MMR IHC tests (98.2%) and only 9 MSI/pMMR cases were identified (2.4%). These results were concordant with recent series (Lindor et al., 2002; Watson et al., 2007; Fazzalari, 2017; Cohen et al., 2018; Jaffrelot et al., 2019; De Salins et al., 2021). A recent work showed that ∼6% of MSI cases retained mismatch repair protein expression and would consequently be missed by IHC testing alone, thereby hindering patient access to immunotherapy (Hechtman et al., 2020). The majority of these cases harbor germline or somatic mismatch repair gene missense mutations; as a result, inactive mutant proteins remain detected by IHC (Peltomäki and Vasen, 2004). Consistently with previous studies, MSI CRCs were associated with female sex, proximal tumor sites, poorly differentiated tumors, and *BRAF* mutation (Tougeron et al., 2015).

In this study, we based our technical exploration of HSP110 on previous publications. For instance, Kim *et al.* developed specific IHC interpretation of HSP110 wt staining based on 3 different scores (Kim et al., 2014). We reproduced the same IHC technique and compared our results with theirs. We highlighted the high percentage of intra-tumor heterogeneity of HSP110 staining in MSI CRC (74.0%), which is similar to the Oh *et al.* study (81%) (Oh et al., 2017). It could be interesting to study whether HSP110 IHC heterogeneity is the reflection of molecular heterogeneity, and if so, whether it is the consequence of the variable deletion size of *HSP110 T*_*17*_ or of post-transcriptional modifications. Nevertheless, despite the intra-tumor heterogeneity, HSP110 groups were comparable between ours and the two published studies with 21.3, 19, and 24% low expression of HSP110 and 78.7, 81, and 76% high expression of HSP110, respectively (Kim et al., 2014; Oh et al., 2017). Since low HSP110 expression is observed in a part of dMMR/MSI CRCs with large *HSP110 T*_*17*_ deletion, while high HSP110 expression is observed not only in normal tissue and in pMMR/MSS CRCs but also in most dMMR/MSI CRCs with small *HSP110 T*_*17*_ deletion, HSP110 expression is not relevant as a diagnostic tool of dMMR/MSI status.

In our cohort, 94.2% of the MSI CRCs had *HSP110 T17* deletion. This percentage was lower than in the Collura *et al.* study (97%) but equivalent to the Berardinelli *et al.* study (94%) and higher than the 88% obtained by Kim *et al.* (Collura et al., 2014; Kim et al., 2014; Berardinelli et al., 2018). The difference with Duval *et al.* and Berardinelli *et al.* results could partly be explained by their use of a ratio calculation method. This quantitative method consisted, in the absence of a clear *HSP110 T17* deletion peak, of calculating ratios of the heights of peaks from the Taq polymerase stutter located at -1 and -2 bp of the polymorphic zone (Buhard et al., 2016). We chose not to use this calculation method because more than 5% of MSS CRC cases explored during our technical development of the *HSP110 T17* deletion test would have then presented *HSP110 T17* deletion. In addition, it was uneasy to clearly discriminate the peak corresponding to the deleted fragment from the stutter peaks of the Taq polymerase. Indeed, discrepancy among different studies could be explained by the difficulties of determining *HSP110 T17* deletion size. Enrichment techniques have recently been described, including modification of the denaturation temperature during the PCR step, which may improve the sensitivity of the analysis of *HSP110 T17* deletion (Baudrin et al., 2018a; Baudrin et al., 2018b; How-Kit et al., 2018).

*HSP110 T*_*17*_ successfully identified 4 cases that were wrongly classified MSI by standard procedure. Our study had not a MSS/pMMR group to determine the specificity of *HSP110 T*_*17*_ deletion in detection of dMMR/MSI CRC. Nevertheless, as previously mentioned, determination of small *HSP110 T*_*17*_ deletion (–1 or –2 bp) is complex, and we recorded some false negative results. Considering all of these limitations, the Pentaplex panel seems easier and more apt than *HSP110 T*_*17*_ deletion to determine MSI status. Buhard *et al.* suggested that compared with the Pentaplex panel, *HSP110 T*_*17*_ deletion showed better sensitivity (0.984 versus 0.951) and similar specificity (0.997) for MSI detection (Buhard et al., 2016). It is worth noting that in this study, only MSI test was performed and not both molecular and MMR IHC. In the Berardinelli *et al.* study, most cases underwent both MSI and MMR IHC tests and eight cases had discordant status (Berardinelli et al., 2018). Finally, *HSP110 T*_*17*_ could be interesting, not to replace MSI and MMR IHC testing, but rather as a complement to the five microsatellites used in the Pentaplex panel to improve detection of MSI cases. Moreover, this marker may be of major interest to investigate discordant cases (dMMR/MSS or pMMR/MSI).

Our study confirmed the correlation between HSP110 wt expression and *HSP110 T*_*17*_ deletion size, first pointed out by Kim et al. (2014). In addition, there was a correlation between the presence of nuclear staining and the deletion size of *HSP110 T*_*17*_ using a cut-off at 4 bp. For the first time, we identified a correlation between *RAS* mutations with both high HSP110 expression and small *HSP110 T*_*17*_ deletion. *RAS* mutations are rare in dMMR/MSI CRC as compared to pMMR/MSS tumors and we have no explanation concerning the association between *RAS* mutations with *HSP110 T*_*17*_ deletion size and HSP110 expression level. *RAS* testing is an important biomarker to predict sensitivity of anti-EGFR in both pMMR/MSS and dMMR/MSI metastatic CRC. However, by contrast to *BRAF* mutation, *RAS* mutation is not a prognosis factor in dMMR/MSI (Rimbert et al., 2017; Tougeron et al., 2020).

Survival and recurrence rates observed in our series were in accordance with previously published studies on dMMR/MSI CRCs (Ribic et al., 2003; Tougeron et al., 2015; Tougeron et al., 2016; Tougeron et al., 2020). Kim *et al.* assessed HSP110 IHC on 168 MSI CRC tissues, among which 167 were analyzed for *HSP110 T*_*17*_ deletion. Associations with clinicopathological, molecular, and survival parameters were statistically analyzed and Kim *et al.* found that low expression of HSP110 was significantly associated with better DFS in the whole cohort, in 5-FU–based adjuvant chemotherapy-treated patients and in the stage III/IV cohort. Analyzing stage III/IV simultaneously is questionable. Besides, the authors did not look specifically at stage II/III patients who received adjuvant 5-FU–based chemotherapy. Moreover, they did not reproduce these results in their second study, in which no correlation was found in either stage II/III patients who received fluoropyrimidine-based adjuvant chemotherapy or in the stage II/III patients who received a combination of 5-FU and oxaliplatin (Kim et al., 2015). In our series, we did not find an association between HSP110 expression and TTR, DFS, OS, or CSS in patients with MSI stage II and III CRC, regardless of whether or not they received adjuvant chemotherapy.

Large deletion of *HSP110 T*_*17*_ has been associated with efficacy of adjuvant chemotherapy (relapse-free survival, RFS), 5-FU alone or with oxaliplatin, in a cohort of 329 patients with stage II and III dMMR/MSI CRCs (Dorard et al., 2011; Collura et al., 2014). It is worth noting that in elderly patients with dMMR/MSI CRC, RFS or DFS is not the most relevant endpoint since some elderly patients could die from other diseases and since dMMR/MSI is associated with good prognosis (Tougeron et al., 2016). TTR seems most relevant. In our series, we did not found any correlation between large *HSP110 T*_*17*_ deletion and TTR/DFS in stage II and III patients receiving adjuvant chemotherapy. Similar to us, no significant survival difference between large and small *HSP110 T*_*17*_ deletions was observed in the Kim *et al.* study (Kim et al., 2014). In addition, only 39% of patients (n = 30/77) were treated with the adjuvant 5-FU plus oxaliplatin regimen in the Collura *et al.* cohort versus 80.6% (*n* = 83/103) in our study (Collura et al., 2014). This difference could be explained by the fact that 5-FU plus oxaliplatin became the standard adjuvant regimen in CRC only in 2004. Regarding these discordant results, HSP110 IHC score or size of *HSP110 T17* deletion are neither robust prognostic biomarkers nor predictive factors of adjuvant chemotherapy efficacy in dMMR/MSI CRC.

Despite its retrospective nature and the prolonged period of the patient’s inclusion, there were few missing clinical data (10%) in our series. In addition, recent prognostic factors were available, like *RAS* and *BRAF* mutations and VELIPI status. One limitation of our study is that we performed a pooled analysis of patients with stage II and III MSI CRC for survival analyses to obtain greater statistical power; however, other series did the same (Collura et al., 2014; Kim et al., 2014). A meta-analysis of all series on HSP110 in MSI CRCs should be performed to confirm the absence of prognostic impact or predictive impact on response to chemotherapy. Up until now, our series is the largest published on MSI CRC patients with simultaneous molecular and IHC analyses of HSP110.

In conclusion, we observed a strong correlation between deletion size of *HSP110 T17* and expression of HSP110. While the *HSP110 T*_*17*_ microsatellite had high sensitivity to detect dMMR/MSI CRC, false negative cases existed. Even though the gold standard for determination of MSI status remains a combination of Pentaplex panel and MMR IHC, the *HSP110 T*_*17*_ microsatellite could help to classify discordant cases. In our series, as in others, we did not identify HSP110 expression or size of *HSP110 T*_*17*_ deletion as prognostic markers or predictors of adjuvant chemotherapy efficacy in MSI CRC. Based on our series and the literature, input of HSP110 for diagnosis or prognosis of dMMR/MSI CRC seems low.

## Data Availability

The original contributions presented in the study are included in the article/Supplementary Material; further inquiries can be directed to the corresponding author.

## Abbreviations

Bp, base pairs; CRC, colorectal cancer; CSS, cancer-specific survival; DFS, disease-free survival; dMMR, deficient mismatch repair system; HSP, heat shock proteins; IHC, immunohistochemistry; MSI, microsatellite instability; OS, overall survival; pMMR, proficient MMR; STAT3, signal transducer and activator of transcription 3; TTR, time to recurrence; VELIPI, vascular emboli or lymphatic invasion or perinervous invasion; wt, wild-type.
