# Supplementary material for: HSP110 as a Diagnostic but Not a Prognostic Biomarker in Colorectal Cancer With Microsatellite Instability
Source: Front Genet. 2022 Jan 3;12:769281. doi: 10.3389/fgene.2021.769281 (PMC8762103; doi:10.3389/fgene.2021.769281)
Supplement: Supplementary file 1 [file Table1.DOCX]

**Supp Table 1. Determination of HSP110 expression by immunochemistry**

| **IHC HSP110 (n=343)** | **Most represented score (%)** | **Second most represented score (%)** | **Highest intensity**  **score (%)** |
| --- | --- | --- | --- |
| **0** | 45 (13.1) | 46 (13.4) | 13 (3.8) |
| **1** | 28 (8.2) | 81 (23.6) | 33 (9.6) |
| **2** | 84 (24.5) | 120 (35.0) | 74 (21.6) |
| **3** | 186 (54.2) | 96 (28.0) | 223 (65.0) |

IHC: Immunohistochemistry
