# Supplementary figures and images for: HSP110 as a Diagnostic but Not a Prognostic Biomarker in Colorectal Cancer With Microsatellite Instability
Source: Front Genet. 2022 Jan 3;12:769281. doi: 10.3389/fgene.2021.769281 (PMC8762103; doi:10.3389/fgene.2021.769281)

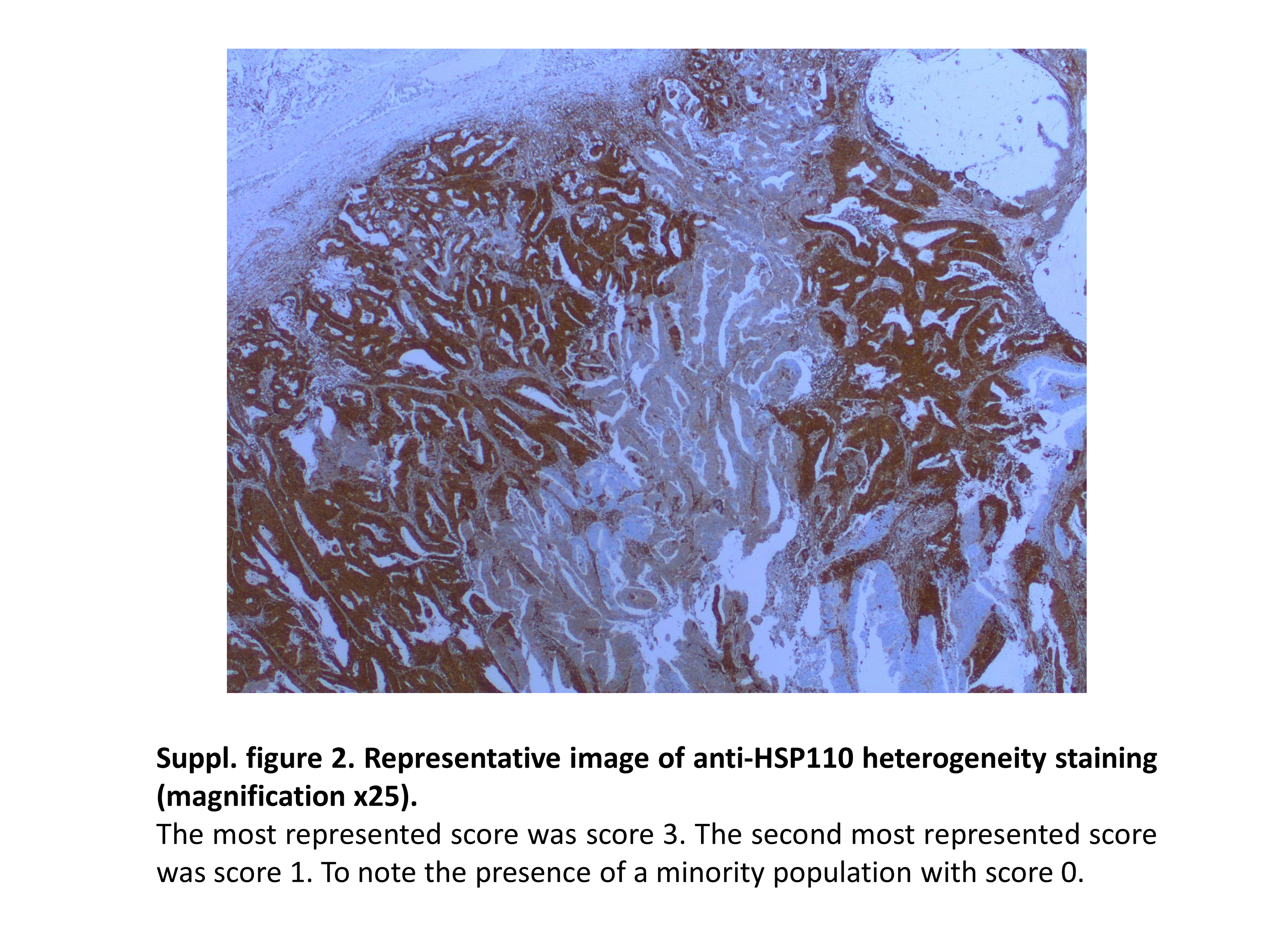

Supplement: Supplementary file 2 [file Image2.tif]

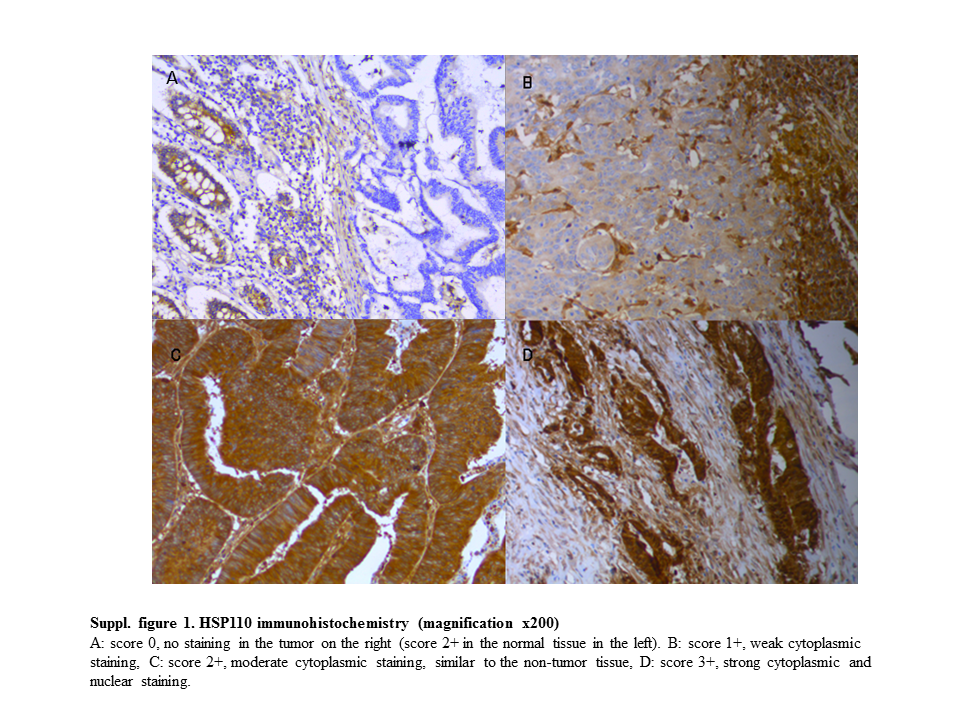

Supplement: Supplementary file 3 [file Image1.tif]
